# MRI Findings of Eosinophilic Fasciitis

**DOI:** 10.5334/jbsr.2805

**Published:** 2022-05-24

**Authors:** Michaela Kubincová, Filip M. Vanhoenacker

**Affiliations:** 1 Department of Radiology, AZ Sint Maarten, Mechelen, Belgium; 2 Department of Radiology, UZ Brussel, VUB, Brussel, Belgium; 3 Faculty of Medicine and Health Sciences, UZ Gent/Antwerp, Belgium; 4 University Hospital Antwerp, Belgium

**Keywords:** eosinophilia, oedema, eosinophilic fasciitis, MRI, skin biopsy

## Abstract

**Teaching point:** Eosinophilic fasciitis (EF) is a rare sclerodermiform disease characterized by upper- and lower-limbs oedema and hardness, which should be confirmed by skin biopsy and MRI in case of clinical suspicion.

## Case

**Figure 1:**
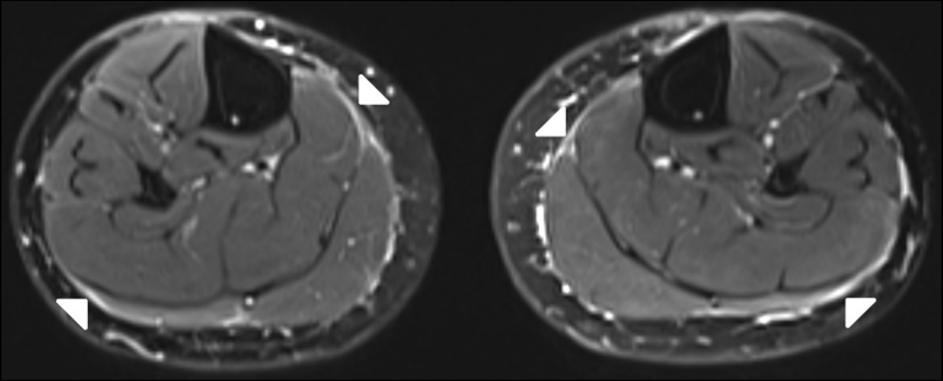


**Figure 2:**
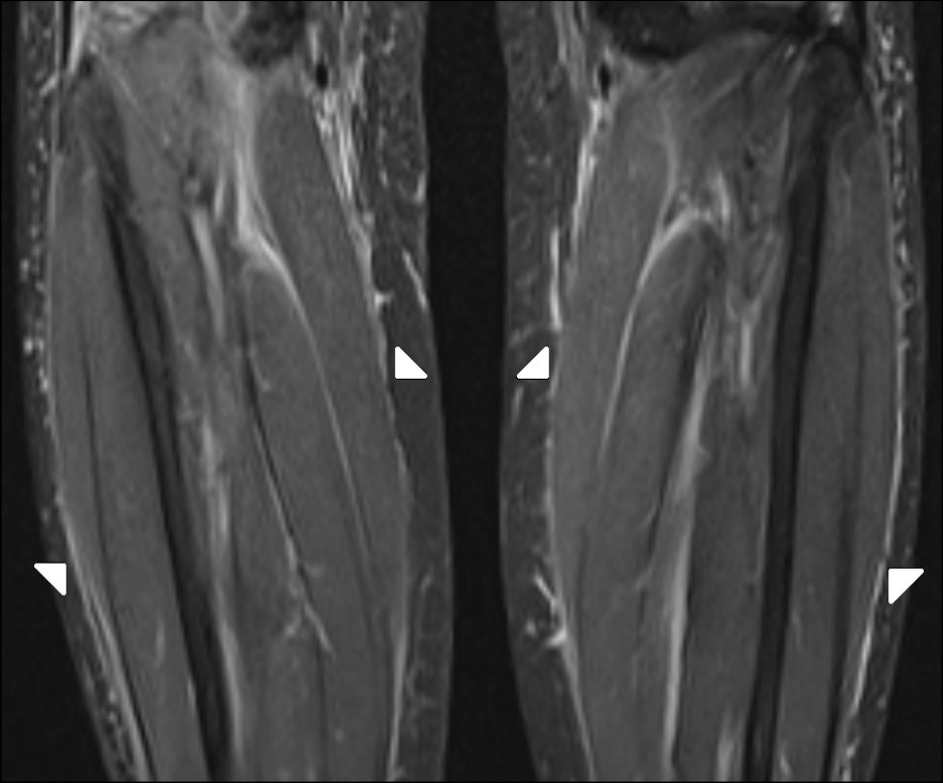


**Figure 3:**
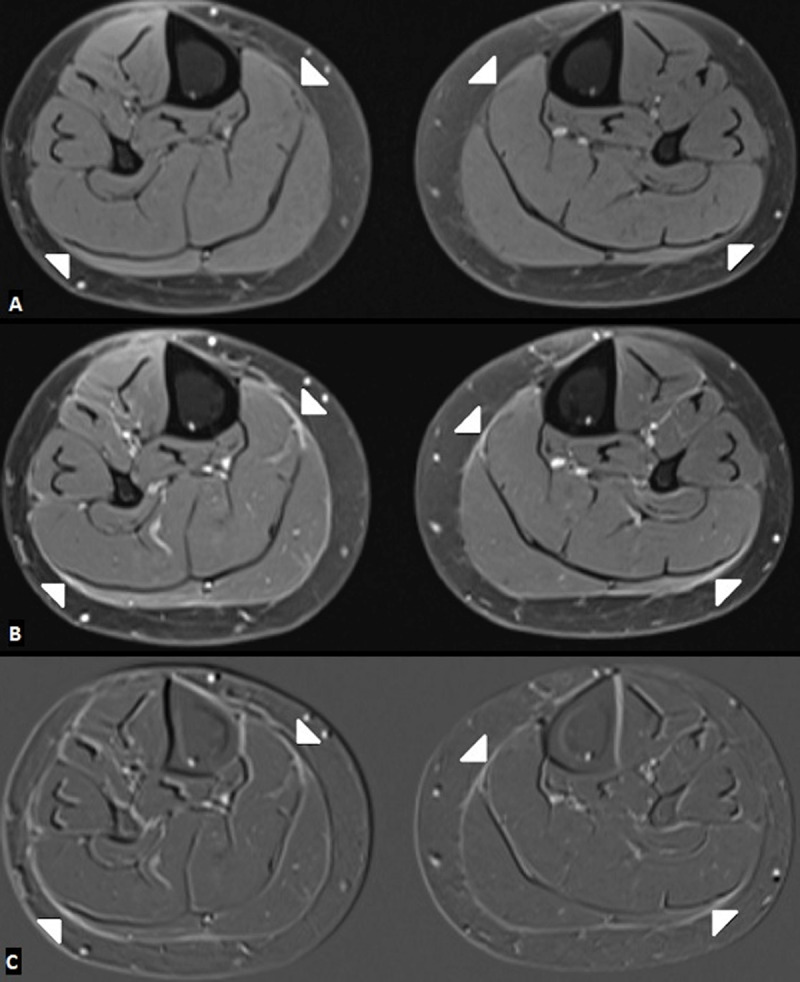


A 64-year-old female with previous history of saphenectomy presented with bilateral new onset symmetric oedema of the upper and lower limbs after the antihypertensive therapy. Laboratory examination showed peripheral eosinophilia. Magnetic resonance imaging (MRI) of the lower limbs demonstrated slightly thickened crural and intermuscular fascia on axial and coronal fatsuppressed (FS) T2-weighted images (WI) (***Figure 1***–***2***; white arrows). The fascia showed enhancement on FS T1-WI (***Figure 3B***, white arrows) and substraction images (***Figure 3C***, white arrows) compared to the non-contrast FS T1-WI (***Figure 3A***; white arrows). The diagnosis of EF was made with skin biopsy from right forearm, which showed diffuse chronic fibrosing fasciitis. The patient was treated with corticosteroids and physiotherapy followed by immunosuppressive therapy because of incomplete response to corticosteroids.

## Comment

EF (Shulman syndrome) is a rare, presumably autoimmune disorder caused by physical exercise, infections, or medication, occurring at any age with slight male predominance. The main clinical symptoms are symmetrical, full-circumference swelling and plate-like hardness of the forearms and lower legs, which may extend to the upper arms and thighs. There is sparing of the fingers and face. There may be concomittant redness and pain. Systemic symptoms are fever or generalized fatigue. Other symptoms include restricted range of joint motion, flexion contracture of the fingers and carpal tunnel syndrome. Patients may become unable to sit on the floor with their buttocks on their heels. The key laboratory findings consist of eosinophilia, elevated sedimentation rate and hypergammaglobulinemia. Elevated serum aldolase and serum type III procollagen peptide levels reflect the disease activity. The gold standard for confirming the presumptive clinical diagnosis is skin biopsy from the skin to the fascia showing marked fascial thickening with inflammatory cell infiltration [1].

MRI is very useful additional diagnostic tool and is used to assess best biopsy site, monitor the course and treatment response. MRI typically demonstrates bilateral symmetric thickening of the fascia on T2-WI and enhancement on post contrast T1-WI. Localized scleroderma is difficult to differentiate from EF. Localized scleroderma presents as unilateral or bilateral skin thickening and hardening with a well-defined border spreading to the fingers. Fibrosis and inflammation in scleroderma typically extend from dermis to fascia and may involve muscle and bone. It may be associated with digital ulcers, Raynaud phenomenon and joint contractures. Treatment consists of corticosteroids. Immunomodulators and physiotherapy for joint contractures. The overall prognosis is good in case of early diagnosis.
